# Multi-homed abnormal behavior detection algorithm based on fuzzy particle swarm cluster in user and entity behavior analytics

**DOI:** 10.1038/s41598-022-26142-w

**Published:** 2022-12-26

**Authors:** Jingyang Cui, Guanghua Zhang, Zhenguo Chen, Naiwen Yu

**Affiliations:** 1 School of Information Science and Engineering, Hebei University of Science and Technology, Shijiazhuang, China; 2 Topsec Network Technology Inc., Beijing, China; 3 Hebei IoT Monitoring Engineering Technology Research Center, North China Institute of Science and Technology, Langfang, China

**Keywords:** Computer science, Information technology

## Abstract

User and entity behavior analytics (UEBA) is an anomaly detection technique that identifies potential threat events in the enterprise's internal threat analysis and external intrusion detection. One limitation of existing methods in UEBA is that many algorithms use deterministic algorithms only for one category labeling and only compare with other samples within this category. In order to improve the efficiency of potential threat identification, we propose a model to detect multi-homed abnormal behavior based on fuzzy particle swarm clustering. Using the behavior frequency-inverse entities frequency (BF-IEF) technology, the method of measuring the similarity of entity and user behavior is optimized. To improve the iterative speed of the fuzzy clustering algorithm, the particle swarm is introduced into the search process of the category centroid. The entity's nearest neighbor relative anomaly factor (NNRAF) in multiple fuzzy categories is calculated according to the category membership matrix, and it is combined with boxplot to detect outliers. Our model solves the problem that the sample in UEBA is evaluated only in one certain class, and the characteristics of the particle swarm optimization algorithm can avoid clustering results falling into local optimal. The results show that compared with the traditional UEBA approach, the abnormal behavior detection ability of the new method is significantly improved, which can improve the ability of information systems to resist unknown threats in practical applications. In the experiment, the accuracy rate, accuracy rate, recall rate, and F1 score of the new method reach 0.92, 0.96, 0.90, and 0.93 respectively, which is significantly better than the traditional abnormal detections.

## Introduction

In the context of globalization, the network security situation of enterprises is complex and changeable, and the rapid development of the Internet faces more network security problems. Cyber security incidents that utilize business design or code flaws to achieve network intrusion and steal data frequently occur, resulting in bad social impacts^1^. While intrusion detection products based on machine learning are widely used, the complexity of business process design and implementation makes it difficult for conventional security products and technical ways to solve problems of the present. Security products such as intrusion prevention systems and intrusion detection systems^2,3^ generate a large number of logs and alarm information every day, and it is difficult to analyze all alarm information one by one. However, actual threatening behaviors such as external attacks and sensitive data leakage are often hidden behind many alarms, which are prone to false negatives and positives, affecting the judgment of analysts^4^.

In order to better detect potential threats and find security problems more accurately, User and Entity Behavior Analytics (UEBA) is proposed^5^. UEBA is developed on the basis of User and Behavior Analytics (UBA) and Security Information and Event Management (SIEM)^6^, and it’s a threat detection method that comprehensively evaluates the risks faced by the system through multi-dimensional^7^. The new Entity object (in UEBA) emphasizes the importance of device behavior in network attack and threat detection. Compared with traditional detection methods, UEBA further improves the accuracy and efficiency of threat detection and increases the expression function of risk judgment, which is beneficial to the system to discover unknown risks and enhance system security^8^.

**Figure 1:**
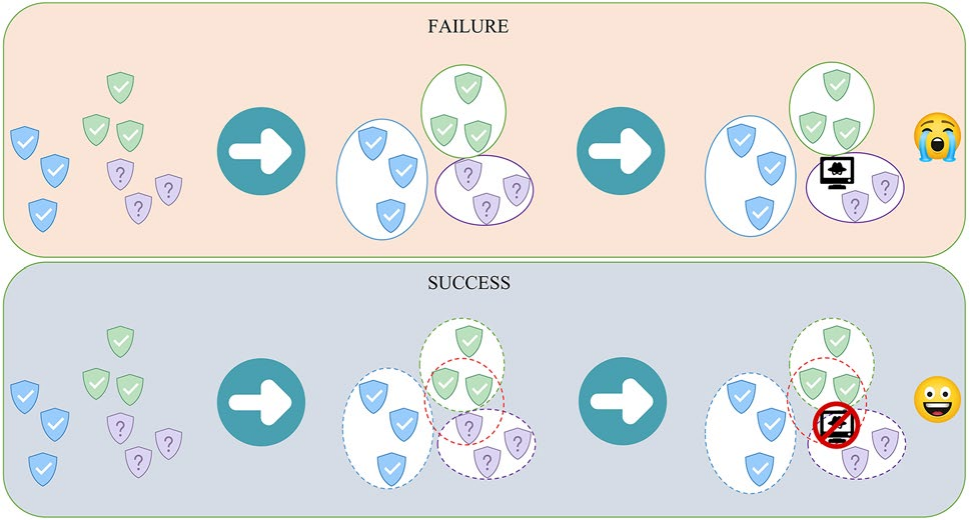
An illustration of our motivation. Compared with the traditional deterministic clustering method, fuzzy clustering for the user and entity behavior is more indispensable in potential abnormal behavior detection.

With the increasing amount of data, the work of labeling samples has become more and more difficult. Currently, some scholars use unsupervised methods for UEBA research by clustering samples together and identifying outliers, such as K-Means^9^ and DBSCAN^10,11^. Gu et al.^12^ proposed a semi-supervised clustering algorithm for optimizing the result of DDoS detection, which can solve the problem of the result easily falling into local optimum by improving the selection of the initial center of the cluster. The model uses the K-Means algorithm to perform cluster analysis on the data, and then the clusters with a small number of elements are regarded as outliers, just like other anomaly detection models based on unsupervised clustering algorithms. In fact, it is not enough to use the clustering algorithm to identify abnormal samples. The clustering results should be combined with other detection methods to improve the detection accuracy. Waqas et al.^13^ proposed a graph clustering algorithm to solve the fraud detection problem in the email sending process. First, the email sending behavior of entities in the dataset was processed through graph clustering to obtain multi-user personalized communities. The dynamic changes in community network structure are one of the important indicators for detecting fraudulent accounts in email systems. However, considering the problem in Fig. 1, the top section does not detect the threat, while the bottom section does. May the samples need to be compared and analyzed in multiple dimensions, because many complex entity behaviors are hidden in normal samples. How to perform the multi-angle comparison of samples and detect threat behaviors hidden in a large number of samples is a significant but less researched issue.

The limitations of the current UEBA method motivated us to conduct this study. This paper proposes a novel *Multi-homed Abnormal Behavior Detection Algorithm based on Fuzzy Particle Swarm Cluster* (MAD-FPC) for User and Entity Behavior Analytics problems. The core mechanism behind MAD-FPC is to use fuzzy clustering algorithms to solve the problem of the lack of sample analytical perspectives in the process of abnormal user and entity behavior detection.

Compared with other UEBA algorithms, the main goal of our proposed MAD-FPC algorithm is to improve the detection effect of abnormal traffic data, and analyze traffic samples from multiple angles, so as to achieve a more accurate identification effect. The experimental results on the NSL-KDD dataset show that the FPC algorithm has fewer iterations compared with the FCM algorithm, and the MAD-FPC algorithm has better anomaly detection metrics compared with other algorithms (LOF, K-Means, Random Forest, One-Class SVM, and kNN). The FPC algorithm goes through two steps: the solution process of the particle swarm, and the calculation process of the membership degree. The time complexity of the particle swarm solution process is related to the number of particles *p*, the number of iterations *q*, and the calculation time *t* of each particle position, so the time complexity of the particle swarm solution process is *O*(*p × t × q*). The particle position needs to be calculated in combination with the fitness function, and the direct cosine similarity between each sample *n* and each class centroid *m* needs to be calculated, so time complexity of algorithm *O*(*t*) = *O*(*n × m*). In the process of calculating the membership degree of fuzzy categories, it is related to the number of samples *n* and the number of categories *m*, and each sample needs to calculate the membership degree of each category, so the time complexity of the fuzzy process of clustering results is *O*(*n × m*). So the time complexity of the FPC algorithm is *O*(*p × n × m × q*) + *O*(*n × m*). Therefore, in the case of large sample size, the time complexity of the FPC algorithm is *O(n* × *m)*. In the MAD algorithm, the degree of anomaly of a sample is related to its *k* nearest neighbors in *m* fuzzy classes. If there are a total of *n* samples, the time complexity of the anomaly detection process is *O*(*n × m × k*). Since MAD-FPC has two processes of clustering and anomaly detection, the time complexity of the MAD-FPC algorithm is *O*(*n × m*) + *O*(*n × m × k*). Since *m* and *k* are constants, in the case of a high number of samples, the time complexity of MAD-FPC is *O*(*n*). When dealing with massive traffic data, it is still necessary to deploy an online detection model in a way of modeling first and then detecting, which is also a common practice of existing network security equipment. First, find the category centroid through historical data, then process the real-time data stream, calculate the membership degree of traffic samples to each category, and then apply the multi-attribution anomaly detection algorithm to calculate the anomaly factor of the sample. When the traffic volume is low, the data clustering centroids can be retrained without affecting the actual business operation. This method of first modeling and then detecting can realize real-time detection of abnormal traffic data.

The main contributions of our work are as follows:

1. We propose a fuzzy particle swarm clustering algorithm, which uses the fuzzy membership matrix to express the possibility of belonging to the class and solves the problem that the fuzzy clustering results easily converge to the local optimum using Swarm Intelligence.
2. The fuzzy clustering results are analyzed using the relative structural relationship among the categories, and a structural anomaly evaluation index NNRAF is proposed to measure the abnormality of samples. This method can refine the measure of the degree of difference among samples and improve the ability to express abnormal situations.
3. A multi-homed abnormal behavior detection model based on fuzzy particle swarm clustering (MAD-FPC) is constructed in detail and complete, which can better solve the problem of abnormal data identification in UEBA.

The rest of this paper is organized as follows. In the “Related works” section, some related works are introduced. And “Methodology” section describes the feature engineering approach to the data, as well as the newly proposed fuzzy clustering and anomaly detection procedures. “Results and discussion” section includes experimental parameter settings, analysis and discussion of experimental results, and comparisons of evaluating performance with other algorithms. The “Conclusion” section briefly summarizes the full text and makes further prospects for future work.

## Related works

### User and entity behavior analytics

UEBA is used to track and monitor some entities' abnormal behaviors, including users, IP addresses, hosts, etc., and can analyze potential malicious activities through contextual correlation of behaviors. Due to the lack of attack methods and the limit of detection capabilities, the target of threat detection was mostly abnormal rule matching^14,15^. The identification logic converted by expert experience is the primary method to detect abnormal behavior^16^, and the results were mostly in two states of "normal" and "abnormal". Later, new attack methods continued to increase, and new threat types continued to emerge, so researchers began to use other methods to identify unknown threats, such as evolutionary algorithms^17^, support vector machine^18^, knowledge graph^19^, etc.

### Fuzzy C-means

FCM^20^ is a commonly used fuzzy clustering method that calculates the membership degree and the centroid of the category through multiple iterations to achieve the maximum intra-class similarity and the minimum inter-class similarity. In the UEBA problem, fuzzy clustering can assign the behavior of an entity to different categories, and a membership matrix expresses the relationship between samples and categories. Although the entity behavior analytics methods based on FCM optimize the results of entity behavior clustering, there are still problems in the process of using the FCM algorithm in UEBA, for example, the initialization parameters are difficult to select, and the results of clustering are easy to fall into the local optimum^21^. In order to better improve the clustering effect of the FCM algorithm, Wang et al.^22^ proposed a validity function to evaluate the clustering results according to the *relative structure information* of the data, and this method can accurately obtain the optimal number of clusters. Wu et al.^23^ added a sample feature called *local data densit*y to optimize the FCM algorithm, and one-dimensional data was extracted by ﻿distribution density characteristics of the eigenvalues. Wang^22^ and Wu^23^ gave us much inspiration for dealing with the relative positional relationship of data, and the shortcomings of FCM in finding the optimal solution cannot also be ignored. Therefore, we added a population-based stochastic optimization technique to the process of finding the centroid and a relative structure measurement method to the anomaly detection process.

### Particle swarm optimization

PSO^24^ is an evolutionary search technology that finds the global optimal solution through repeated particles jumping. During the execution of PSO, all particles are assigned initial random positions and initial random velocities. Then the speed and direction of each particle are calculated by parameters initialized earlier, including the global optimal position and the optimal individual position. With continuous iterations, particles gather around one or more optimal points by exploring and exploiting existing vantage points in the search space. By considering the global optimal position and its own optimal position in the moving direction, PSO can avoid prematurely falling into the local optimal solution^25^. The genetic algorithm can be used as the focus of finding cluster centers, and Chicco et al.^26^ proposed an ant colony clustering to evolve the centroids of sets in the iterative process. We refer to this idea to optimize the process of finding fuzzy cluster centers.

### Anomaly behavior detection

The abnormal behavior detection in the UEBA field mainly consists of external intrusion and internal threat detection. In terms of external intrusion identification, Pan et al.^27^ designed a dynamic residual generator to detect a variety of attack methods through an entity behavioral analysis filtering device, which solved the problem of a few types of abnormal detection in static detection models. Some scholars have also established an anomaly detection model to detect Cyber attacks based on CNN^28,29^, RNN^30^, long short-term memory (LSTM)^31–33^, GAN^34,35^, and deep autoencoder (DAE)^36,37^. In terms of insider threat identification, the research focuses on the analysis of enterprise employee behavior^38^, user portrait^39,40^, complex behavior modeling^41^, and so on. Intranet users often have very high system permissions, so their operations often have a more significant impact. Once an abnormal situation occurs, it may cause extremely serious losses. Therefore, the next stage of UEBA's research may focus on the analytics of enterprise employee behavior, which we considered as well.

However, most anomaly detection methods use a "hard division" strategy when clustering samples^42^. Each sample belongs to only one category, so one sample can only be compared with other samples within this category when performing anomaly calculations. The behavior of users and entities is *not only complex but also camouflaged* and *should be analyzed in comparative experiments as much as possible*. The correlation information between samples and various clusters can be well preserved if we use the fuzzy clustering algorithm to analyze user and entity behavior data. The threat degree of users and entities can be judged through multi-angle evaluation, so as to obtain more accurate abnormal detection results^43^.

## Methodology

**Figure 2:**
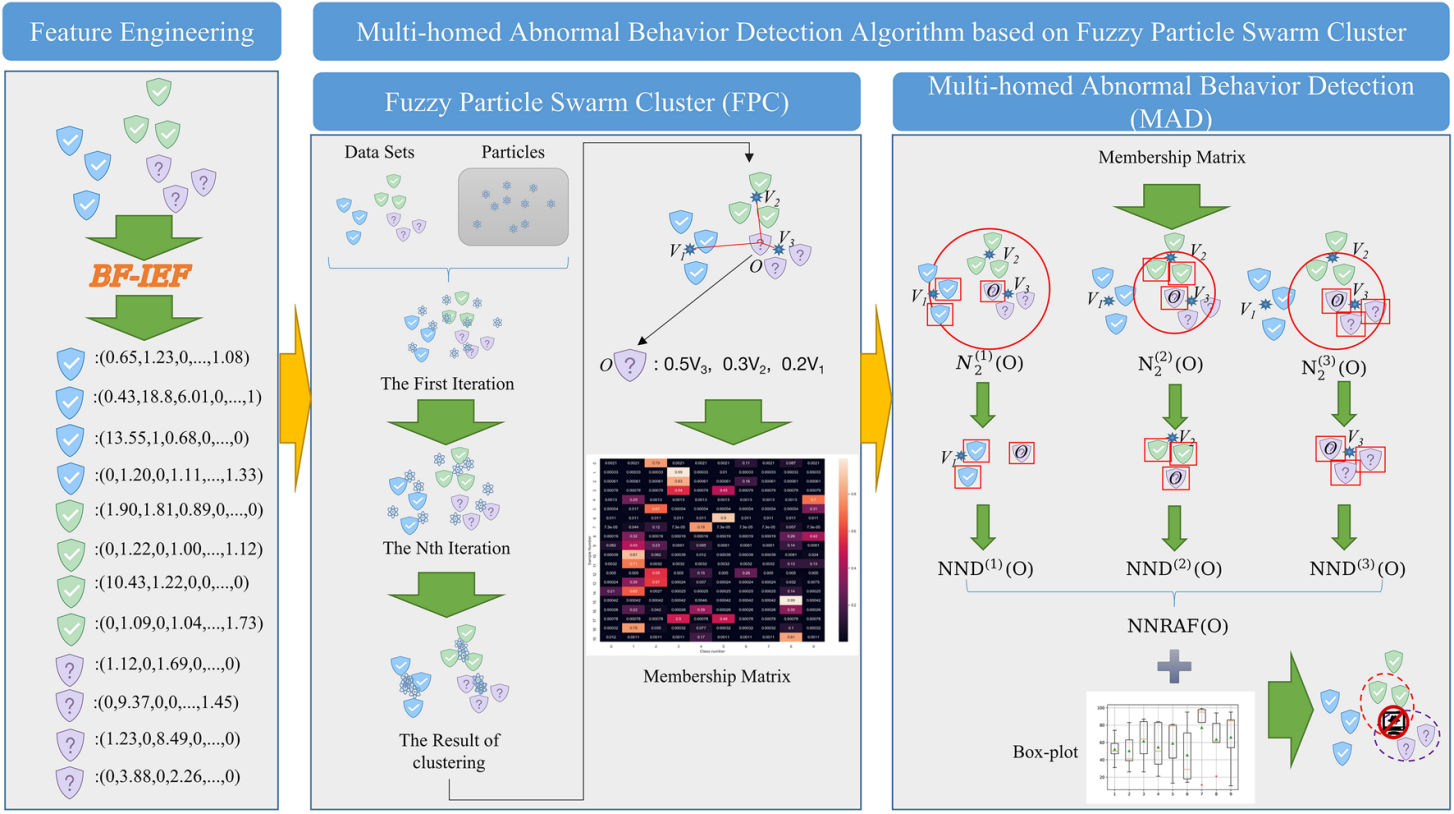
An overview of our model. The overall processing process is divided into three parts, in the feature engineering phase, BF-IEF is mainly used to convert traffic data into entity behaviors. In the FPC stage, particle swarms are used to search for the clustering results, and the membership matrix is obtained. In the MAD process, we extracted the 2-nearest neighbors of point *O* in three classes, denoted by ${N}_{2}^{(m)}(O)$. After that, the *NND* of point *O* in each class is calculated and combined together to get *NNRAF*. Finally, anomaly detection is performed based on the boxplot.

The overall processing flow of the user and entity behavior anomaly detection algorithm proposed in this paper is shown in Fig. 2, it includes three main parts:

1. Feature engineering.
2. Fuzzy particle swarm clustering (FPC).
3. Multi-homed anomaly behavior detection (MAD).

First, we collect data in the network environment and perform vectorization and standardization operations. Moreover, a feature engineering called BF-IEF is used to derive the appropriateness of the behavior vector. Next, the fuzzy clustering results are obtained through the fuzzy particle swarm fuzzy clustering process, and then the fuzzy clustering results are subjected to multi-homed anomaly behavior detection.

### BF-IEF feature engineering

In the traditional FCM algorithm, the Euclidean distance is mainly used to measure the similarity of vectors between the sample and the class center. But Euclidean distance measures the straight-line distance of each point in space, which is directly related to the position coordinates of each point, while cosine distance measures the angular relationship of vectors in space, which is more reflected in the difference in direction^44^. In the process of fuzzy clustering analytics of user and entity behaviors, we found it is better to use cosine similarity between vectors to distinguish behavior differences, so based on the TF-IDF (Term Frequency-Inverse Document Frequency) idea, we optimized the representation method of entity behavior vectors and used cosine similarity to analyze samples.

TF-IDF is a weighting technique often used in text mining to assess the importance of a word to one of the documents in a document set or corpus. The importance of a word increases proportionally to the number of times it appears in the document and decreases inversely proportional to its frequency in the corpus. The core idea is that if a word appears in an article with a high frequency and rarely appears in other articles, it is considered that the word or phrase has an excellent ability to distinguish the document to which it belongs from other documents.

Based on the idea of TF-IDF, we propose a feature engineering method for user and entity behavior. The method of calculating Behavior Frequency-Inverse Entities Frequency (BF-IEF) is as follows. Given an entity behavior B, the calculation method of entity behavior frequency (BF) of B is:

1 $$BF = \frac{The \,frequency\, of\, B\, in \,all\, behaviors\, of\, this \,entity}{{The\, number \,of\, all \,behaviors\, of\, this\, entity}}$$

The meaning of formula (1) is that if B appears more times in the entity behavior set, it proves that B is the habit of the entity, so that B can distinguish the difference between this entity and other entities very well.

The reverse entity frequency IEF calculation method for behavior B is:

2 $$IEF=\mathrm{log}\left(\frac{The\;total\;number\;of\;entities\;in\;the\;dataset}{Number\;of\;entities\;whose\;behavior\;includes\;B+1}\right)$$

Adding one to the denominator in the formula is to prevent the denominator from being 0. The meaning of formula (2) is that if a behavior appears many times in many entity behavior sets, it proves that many entities are accustomed to doing this behavior, and the dissimilarity between entities cannot be well distinguished by this behavior.

The product of the frequency of the entity behavior and the frequency of the reverse entity is:

3 $$BF-IEF=BF*IEF$$

The meaning of formula (3) is that the more times a behavior appears in an entity's behavior set, and the fewer times it appears in other entities' set, the better it can represent this entity. Using the BF-IEF to process the data, the entity behavior vector can be expressed more accurately, and the clustering algorithm's accuracy can be enhanced simultaneously.

Based on the representation method of entity behavior vector and given the data sample *x* and the cluster category center *v*, the distance between the data sample and the cluster category center can be calculated. The vector similarity of Behavioral Measure (BM) can be calculated by the cosine similarity, and the calculation method is shown in formula (4):

4 $$BM\left({x}_{i},{v}_{j}\right)=\left|x\right|\left|v\right|cos\theta =\frac{{\sum }_{i=1}^{n}({x}_{i}\times {v}_{i})}{\sqrt{{\sum }_{i=1}^{n}{({x}_{i})}^{2}}\times \sqrt{{\sum }_{i=1}^{n}{({v}_{i})}^{2}}}.$$

### Fuzzy particle swarm clustering (FPC)

The fuzzy set was proposed by Prof. A. Zadeh^45^ in 1965 based on fuzzy mathematics, which broke through the method of using inclusion and non-inclusion in classical sets to describe the relationship between elements and sets. Fuzzy sets perform soft division on sets and elements and use appropriate membership functions to describe the relationship between groups and pieces.

In fuzzy clustering, we use the degree of membership to represent the possibility of a sample *x* being included in class *c*, generally denoted as *u*_*ij*_. The *u*_*ij*_ represents the membership degree of the *i*_th_ sample to the *j*_th_ category, whose value range is [0,1]. When *u*_*ij*_ = 0, it means that the *i*_th_ sample must not belong to the *j*_th_ category; when *u*_*ij*_ = 1, it means that the *i*_th_ sample must belong to the *j*_th_ category.

Assuming that the data set $X=\{{x}_{1},{x}_{2},\dots ,{x}_{N}\}$, the clustering category $C=\{{C}_{1},{C}_{2},\dots ,{C}_{C}\}$, the *j*_th_ clustering category center ${v}_{j}$. The objective function of entity behavior Fuzzy Particle Swarm Clustering (FPC) algorithm is shown in formula (5):

5 $${\underset{{u}_{\mathit{ij}},{v}_{j}}{\mathrm{min}}J}_{m}({u}_{ij},{v}_{j})=\sum_{i=1}^{N}\sum_{j=1}^{C}{{u}_{ij}}^{m}{BM\left({x}_{i},{v}_{j}\right)}^{2}$$

Subject to:

6 $$\sum_{j=1}^{C}{u}_{ij}=1$$

In formula (5), *m* is a fuzzy coefficient and the value range of *m* is [1, + ∞). The *m* is a parameter used to adjust the degree of clustering fuzziness. The larger the value of *m*, the more ambiguous the clustering result will be. Generally, most experiments take *m* = 2. It is worth noting that the sum of all membership degrees should be equal to 1, as in formula (6).

Using the Lagrangian multiplier method^46^, the Lagrangian Multiplier is introduced to transform Eqs. (5) and (6) into unconditional extreme value problems:

7 $$\mathrm{min }\mathcal{L}\;\left({u}_{ij},{v}_{j}\right)=\sum_{i=1}^{N}\sum_{j=1}^{C}{{u}_{ij}}^{m}{BM\left({x}_{i},{v}_{j}\right)}^{2}+\sum_{i=1}^{N}{\lambda }_{i}\left(\sum_{j=1}^{C}{u}_{ij}-1\right)$$

By derivation of the variables in formula (7), the extreme points of each variable can be obtained.

Taking the partial derivative for *u*_*ij*_ is equivalent to taking the partial derivative for the first half and the second half of the Langrangian function:

8 $$\frac{\partial \mathcal{L}}{\partial {u}_{ij}}=\frac{\partial }{\partial {u}_{ij}}\left[\sum_{i=1}^{N}\sum_{j=1}^{C}{{u}_{ij}}^{m}{BM\left({x}_{i},{v}_{j}\right)}^{2}+\sum_{i=1}^{N}{\lambda }_{i}\left(\sum_{j=1}^{C}{u}_{ij}-1\right)\right]=\frac{\partial }{\partial {u}_{ij}}\sum_{i=1}^{N}\sum_{j=1}^{C}{{u}_{ij}}^{m}{BM\left({x}_{i},{v}_{j}\right)}^{2}+\frac{\partial }{\partial {u}_{ij}}\sum_{i=1}^{N}{\lambda }_{i}\left(\sum_{j=1}^{C}{u}_{ij}-1\right)$$

Calculate the two parts separately.

9 $$\frac{\partial }{\partial {u}_{ij}}\sum_{i=1}^{N}\sum_{j=1}^{C}{{u}_{ij}}^{m}{BM\left({x}_{i},{v}_{j}\right)}^{2}= \sum_{i=1}^{N}\sum_{j=1}^{C}{{m u}_{ij}}^{m-1}{BM\left({x}_{i},{v}_{j}\right)}^{2}$$

10 $$\frac{\partial }{\partial {u}_{ij}}\sum_{i=1}^{N}{\lambda }_{i}\left(\sum_{j=1}^{C}{u}_{ij}-1\right)=\frac{\partial }{\partial {u}_{ij}}\sum_{i=1}^{N}\left(\sum_{j=1}^{C}{u}_{ij}{\lambda }_{i}-{\lambda }_{i}\right)=\sum_{i=1}^{N}\sum_{j=1}^{C}{\frac{\partial }{\partial {u}_{ij}}u}_{ij}{\lambda }_{i}=\sum_{i=1}^{N}\sum_{j=1}^{C}{\lambda }_{i}=\sum_{i=1}^{N}C{\lambda }_{i}$$

According to formula (9) and formula (10), there are:

11 $$\frac{\partial \mathcal{L}}{\partial {u}_{ij}}=\sum_{i=1}^{N}\sum_{j=1}^{C}{{m u}_{ij}}^{m-1}{BM\left({x}_{i},{v}_{j}\right)}^{2}+\sum_{i=1}^{N}C{\lambda }_{i}$$

Let formula (11) equal zero, then:

12 $$\begin{aligned} & \mathop \sum \limits_{i = 1}^{N} \left( {\mathop \sum \limits_{j = 1}^{C} m u_{ij}^{m - 1} BM\left( {x_{i} ,v_{j} } \right)^{2} + C\lambda_{i} } \right) = 0 \\ & \quad \Rightarrow \mathop \sum \limits_{j = 1}^{C} m u_{ij}^{m - 1} BM\left( {x_{i} ,v_{j} } \right)^{2} + C\lambda_{i} = 0 \\ & \quad \Rightarrow \mathop \sum \limits_{j = 1}^{C} m u_{ij}^{m - 1} BM\left( {x_{i} ,v_{j} } \right)^{2} + \mathop \sum \limits_{i = 1}^{C} \lambda_{i} = 0 \\ & \quad \Rightarrow \mathop \sum \limits_{j = 1}^{C} \left[ {m u_{ij}^{m - 1} BM\left( {x_{i} ,v_{j} } \right)^{2} + \lambda_{j} } \right] = 0 \\ \end{aligned}$$

And because the degree of membership ${u}_{ij}$ is a non-negative value, then ${{m u}_{ij}}^{m-1}{BM\left({x}_{i},{v}_{j}\right)}^{2}$ is also a non-negative value. At the same time, the Lagrange multiplier ${\lambda }_{j}$ is non-negative, then:

13 $${{m u}_{ij}}^{m-1}{BM\left({x}_{i},{v}_{j}\right)}^{2}+{\lambda }_{j}\ge 0$$

Simultaneous formula (12) and formula (13), we can get:

14 $${{m u}_{ij}}^{m-1}{BM\left({x}_{i},{v}_{j}\right)}^{2}+{\lambda }_{j}=0$$

By analyzing formula (14), we can get:

15 $${u}_{ij}={\left(\frac{-{\lambda }_{j}}{m{BM\left({x}_{i},{v}_{j}\right)}^{2}}\right)}^{\frac{1}{m-1}}$$

And we define

16 $$\sum_{k=1}^{N}{u}_{kj}=1 , \forall j=1, 2, \dots , C$$

And based on above, we can derive that:

17 $$\begin{aligned} \mathop \sum \limits_{k = 1}^{N} \left( {\frac{{ - \lambda_{j} }}{{mBM\left( {x_{k} ,v_{j} } \right)^{2} }}} \right)^{{\frac{1}{m - 1}}} & = \left( {\frac{{ - \lambda_{j} }}{m}} \right)^{{\frac{1}{m - 1}}} \mathop \sum \limits_{k = 1}^{N} \left( {\frac{1}{{BM\left( {x_{k} ,v_{j} } \right)^{2} }}} \right)^{{\frac{1}{m - 1}}} = 1 \\ & \Rightarrow \left( {\frac{{ - \lambda_{j} }}{m}} \right)^{{\frac{1}{m - 1}}} = \frac{1}{{\mathop \sum \nolimits_{k = 1}^{N} \left( {\frac{1}{{BM\left( {x_{k} ,v_{j} } \right)^{2} }}} \right)^{{\frac{1}{m - 1}}} }} \\ \end{aligned}$$

Substitute Eq. (17) into Eq. (15) to obtain the calculation formula of the final ${u}_{ij}$:

18 $${u}_{ij}=\frac{1}{\sum_{k=1}^{N}{\left(\frac{1}{{BM\left({x}_{k},{v}_{j}\right)}^{2}}\right)}^{\frac{1}{m-1}}} \cdot {\left(\frac{1}{{BM\left({x}_{i},{v}_{j}\right)}^{2}}\right)}^{\frac{1}{m-1}}=\frac{1}{\sum_{k=1}^{N}{\left(\frac{{BM\left({x}_{i},{v}_{j}\right)}^{2}}{{BM\left({x}_{k},{v}_{j}\right)}^{2}}\right)}^{\frac{1}{m-1}}}={\left[\frac{1}{\sum_{k=1}^{N}\left(\frac{{BM\left({x}_{i},{v}_{j}\right)}^{2}}{{BM\left({x}_{k},{v}_{j}\right)}^{2}}\right)}\right]}^{\frac{1}{m-1}}= {\left[\frac{1}{\sum_{k=1}^{N}{\left(\frac{BM\left({x}_{i},{v}_{j}\right)}{BM\left({x}_{k},{v}_{j}\right)}\right)}^{2}}\right]}^{\frac{1}{m-1}}$$

In order to get better clustering results, we use particle swarm optimization algorithm to optimize the process of finding cluster centers. In the PSO algorithm, the speed and direction of the particles are iterated by their own optimal solution and the global optimal solution, and the determination of the optimal solution is very related to the objective function. In the FPC algorithm, we redesigned the particle structure and objective function, and the particle will have a matrix structure during initialization to store the samples' relative position relationship with the center of each category.

It may be assumed that there are *N* particles in the *D*-dimensional space, and each particle has two characteristics: position characteristics, and velocity characteristics.

Given particles position features:

$$X=({x}_{1},{x}_{2},\dots ,{x}_{n})$$

And particles velocity features:

$$V=\left({v}_{1},{v}_{2},\dots ,{v}_{n}\right)$$

We can get the position and velocity parameters of new particles in the process of population evolution. The calculation formula is:

19 $${v}_{i+1}={v}_{i}+{c}_{1}\times rand\left(a\right)\times \left({pbest}_{i}-{x}_{i}\right)+{c}_{2}\times rand\left(b\right)\times \left({gbest}_{i}-{x}_{i}\right)$$

Subject to:

20 $${x}_{i+1}={x}_{i}+{v}_{i+1}$$

In formula (19), *i* represents the result of the *i*_th_ iteration, ${v}_{i}$ represents the speed of the particle at the *i*_th_ iteration; *rand()* is a random function, and the random value is between (0, 1); ${pbest}_{i}$ represents the optimal position of the particle itself in the *i*_th_ iteration; ${gbest}_{i}$ is the global optimal position in the *i*_th_ iteration; and ${x}_{i}$ is the current position of the particle; ${c}_{1}$ and ${c}_{2}$ are learning factors, by taking ${c}_{1}={c}_{2}=2$. It is worth noting that in the particle swarm algorithm, it is also necessary to set a maximum value of particle velocity to ensure that the search process for the optimal value will not cause the situation that the step size is too large to cause the failure to converge. ${V}_{max}$ is the maximum speed, if ${v}_{i}>{V}_{max}$, then let ${v}_{i}={V}_{max}$.

We take the objective function of fuzzy clustering formula (5) as the fitness function of particle swarm, so as to ensure the judgment of individual optimal and local optimal. After each particle position iteration is completed, the current fitness value is compared with the previous fitness value, and it is determined whether the particle position needs to be updated. The detailed steps of the *Fuzzy Particle Swarm Clustering* Algorithm are as follows:
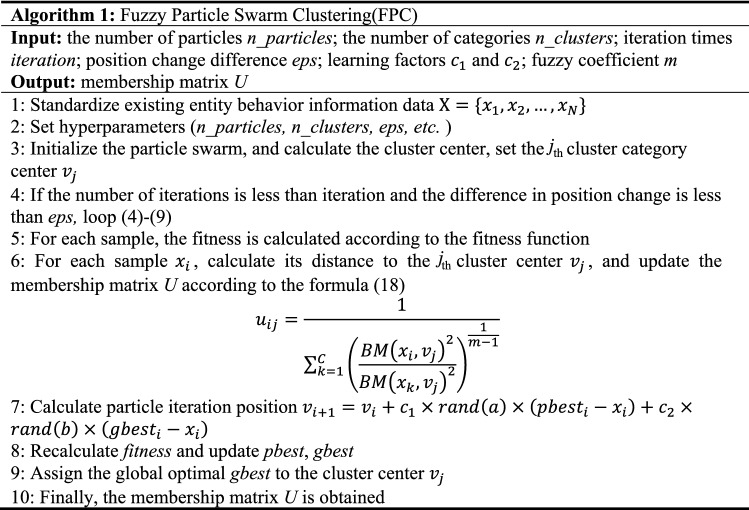


*Algorithm 1* mainly performs the fuzzy clustering process. The optimal clustering point is obtained by repeated jumping of the particle swarm, and the membership relationship between the sample and the category is calculated according to the membership degree formula, and finally the membership degree cluster U is obtained. The next step is multi-homed anomaly detection.

### Multi-homed abnormal behavior detection (MAD)

With the continuous development of network technology, abnormal situations used in entity behaviors show obvious complex and hidden characteristics^47^. Most of the existing anomaly detection algorithms analyze the behavior content based on a single dimension. Using traditional abnormal detection methods to analyze anomalies in the fuzzy clustering results will lose the "*fuzzy*" characteristics of the fuzzy clustering results. The local outlier factor (LOF) algorithm^48^ is a common density-based single-dimensional anomaly detection algorithm. It compares the *local outlier factor* between the node and the neighboring node to determine whether the node is abnormal. Based on the LOF algorithm, this paper designs a *Multi-homed Abnormal Behavior Detection(MAD)* algorithm to solve the anomaly detection problem of fuzzy clustering results.

Among all the data points in the same cluster, finding the *k*_th_ point closest to point *O*, the distance between *this point* and *point O* is called the *K-nearest neighbor distance,* represented by *KNND(O)*. The larger the *KNND(O)*, the sparser the points around *O*, and the further away *O* is from the place where the amount of data is distributed.

It is worth noting that if point *O* belongs to *M cluster*s as the result of fuzzy clustering, the number of *KNND(O)* will be *M*. This means that point O will have *M KNND(O)s*. All points from the *k*_th_ point to point *O* are included in the *k-nearest neighbors* of point *O*, which can be represented by ${N}_{K}^{(m)}(O)$. The subscript *K* represents the number of neighbors, which is input by the user; the superscript *m* represents the neighbors of *O* in the *m*_th_ category, and the value range of *m* is [1, M]. The reachable distance between point *p* and point *O* is defined as the maximum value of "*k-nearest neighbor distance of point O*" and "*direct distance between point p and point O*". The calculation method is as follows:

21 $$reach\_dist\left(p,O\right)=\mathrm{max}\left\{KNND\left(O\right), dist\left(p,O\right)\right\}$$

Another thing worth noting is that the reachable distance is directional, and the reachable distance from point *p* to point *O* may not be equal to the reachable distance from point *O* to point *p*. The *nearest neighbor density(NND)* of point *O* is used to measure the relative density of point *O* and other surrounding points in the same cluster. It is defined as the reciprocal of the average reachable distance between other points in the *K* neighborhood of each cluster and point O, and the calculation method is:

22 $${NND}^{(i)}\left(O\right)=\frac{1}{\frac{{\sum }_{p\epsilon {N}_{K}^{(m)}(O)}reach\_dist\left(p,O\right)}{\left|{N}_{K}^{(m)}(O)\right|}}$$

The superscript *m* represents the *m*_th_ category, and by calculating the *NND* of samples that belong to the cluster of each point *O*, the neighbor density of *O* can be obtained. The lower the average distance, the higher the neighbor density. A high density of neighbors means that the data around the point is denser. In addition, there may be more than *k* points in the neighborhood of point *O*, so the sum of reachable distances should be normalized according to the actual situation.

In the MAD algorithm, we use the point *O*'s *Nearest Neighbor Relative Anomaly Factor (NNRAF)* to measure the degree of abnormal of point *O*, and its anomaly factor score is the ratio of *the average of samples’ NND that are located around O in M categories* to *O’s NND*. The formula for calculating *NNRAF* is

23 $$NNRAF\left(O\right)=\sum_{m=1}^{M}\frac{{u}_{m}{\sum }_{p\in {N}_{k}^{(m)}\left(O\right)}\frac{{NND}^{\left(m\right)}(p)}{{NND}^{\left(m\right)}(O)}}{\left|{N}_{k}^{(m)}\left(O\right)\right|}$$

Among them, *m* represents the *m*_th_ cluster; ${N}_{k}^{(m)}\left(O\right)$ is the point where is within the k nearest neighbors of point *O in* the *m*_th_ cluster; ${NND}^{\left(m\right)}(O)$ is *O*’s *NND* in *m*_th_ category; ${u}_{m}$ is the degree of membership of O to *m*_th_ cluster.

Under normal circumstances, when using LOF for abnormal sample detection, 1 is used as a threshold for comparison. If the LOF of a point is less than 1, it proves that the point is surrounded by dense samples, and this point belongs to the normal category. The opposite is the case for greater than 1. In some datasets with a flat distribution, an *NNRAF* value greater than 0.8 may be an abnormal sample; while in some datasets with large distribution fluctuations, an *NNRA*F value greater than 2 may be a normal sample. Obviously, using a fixed threshold to detect *NNRAF* cannot flexibly handle the actual situation, and since the membership information of multiple clusters is added to the calculation process of the abnormal factor, the value of *NNRAF* changes greatly, so we decided to optimize the detection threshold of the algorithm by the boxplot.

The boxplot was proposed by American statistician John W. Tukey in 1977^49^. It consists of five numerical points: Minimum (Q_0_ or 0_th_ percentile), Maximum (Q_4_ or 100_th_ percentile), Median (Q_2_ or 50_th_ percentile), First quartile (Q_1_ or 25_th_ percentile), and Third quartile (Q_3_ or 75_th_ percentile). IQR means Interquartile Range, calculated by subtracting Q_1_ from Q_3_. Compared with the *3σ Rule*^50^, using the boxplot to detect outliers does not require the data to be normally distributed, which greatly increases the scope of the algorithm. When the boxplot is used for outlier detection, it mainly uses the five statistics in the data mentioned above. The calculation formula of the threshold (*Th*) is:

24 $$Th={Q}_{3}+3{I}_{QR}$$

In general, values greater than *Th* should be flagged as abnormal. Using formula (24) can judge the non-normally distributed data, thereby improving the applicable scope of *NNRAF*. The detailed steps of the *Multi-homed Abnormal Behavior Detection* Algorithm are as follows:
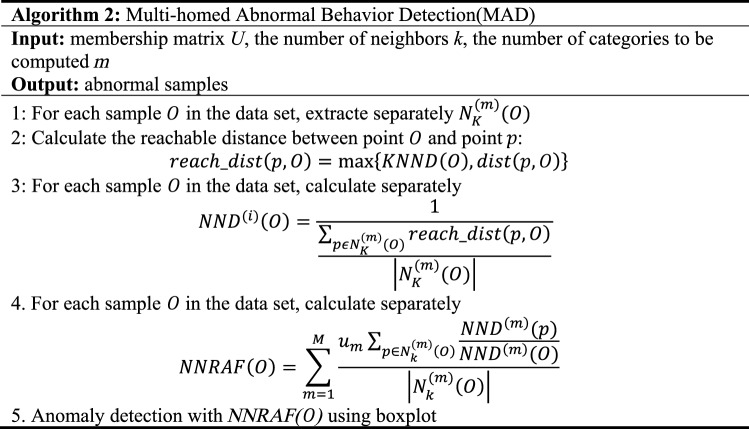


*Algorithm 2* mainly performs the multi-homed anomaly detection process. Calculate its NND in each fuzzy class for each sample, and synthesize them together to get NNRAF, and finally use the boxplot for anomaly identification.

### MAD-FPC flow chart

**Figure 3:**
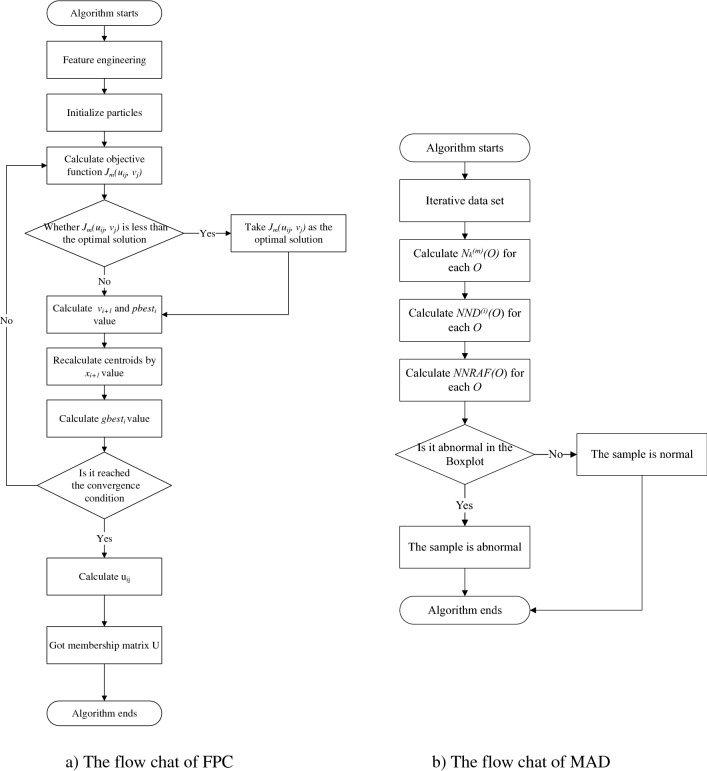
MAD-FPC flow chart.

Based on fuzzy particle swarm clustering algorithm (FPC) and multi-homed abnormal behavior detection algorithm (MAD), a fuzzy-based multi-homed anomaly detection MAD-FPC algorithm is designed. First, the BF-IEF technology is used to normalize and initialize the entity behavior, and then the fuzzy clustering results of each entity behavior are obtained by multiple iterations, and next the relative anomalies of the samples are calculated according to the cluster membership matrix, and finally, the boxplot is used for detection. The algorithm flow is shown in Fig. 3.

## Results and discussion

### Data description and preliminary processing

In order to verify the effectiveness of the anomaly detection algorithm, this paper uses NSL-KDD^51^ data to simulate the connection behavior of entities and users in network communication. We preprocessed the data with feature engineering, and each connection after processing has a total of 43 features. In the process of feature engineering, the character-type discrete data is processed by using One-Hot encoding. In order to better distinguish each connection, we use the BF-IEF weighting technique described in “BF-IEF Feature Engineering” section to concatenate the three features of "Protocol Type", "Service" and "Flag" as user and entity behaviors. In addition, we also use the normalization method to scale other data numerically to compress them between 0 and 1. The normalization method is:

25 $${x}^{^{\prime}}=\frac{x-\mathrm{min}\left(x\right)}{\mathrm{max}\left(x\right)-\mathrm{min}\left(x\right)}$$

NSL-KDD is an improved version of the KDD CUP 99 dataset. It solves the problems of data redundancy and duplication of test and training data in KDD CUP 99 and optimizes the sample ratio to a certain extent. The data distribution of NSL-KDD is shown in Table 1:

**Table 1:** Category distribution of experimental data.

Attack types	KDDTrain^+^		KDDTest^+^	
	Amount	Percentage	Amount	Percentage
Normal	67,343	53.46%	9711	43.07%
DOS	45,927	36.46%	7460	33.09%
Probe	11,656	9.25%	2421	10.74%
U2R	52	0.04%	67	0.30%
R2L	995	0.79%	2885	12.80%
Total	125,973	100%	22,544	100%

We use the KDDTrain^+^ for training and the KDDTest^+^ for testing. This dataset includes 4 types of abnormal traffic, namely DOS, Probe, U2R, and R2L. DOS, which stands for Denial-of-Service, has occupied the largest proportion of abnormal samples. Probe (PROBING) stands for surveillance and probing. U2R (USER-TO-ROOT) is unauthorized access to superuser privileges by a local unprivileged user. R2L (REMOTE-TO-LOCAL) is unauthorized access from a remote host. These four types of anomalies include 38 attack subtypes, and the specific corresponding situations are shown in Table 2.

**Table 2:** Attack categories of NSL-KDD dataset.

Attack types	Attack subtypes
Normal	Normal
DOS	Dos, Apache2, Back, Land, Mailbomb, Neptune, Pod, Smurf, Teardrop, Udpstorm
U2R	U2R, Buffer_overflow, Loadmodule, Perl, Rootkit
R2L	R2L, Ftp_write, Guess_passwd, Imap, Multihop, Named, Phf, Sendmail, Snmpgetattack, Snmpguess, Spy, Warezclient, Warezmaster, Worm, Xlock, Xsnoop
Probe	Probe, Mscan, Nmap, Saint, Portsweep, Ipsweep, Satan

### Evaluation indicators

In the process of evaluating the algorithm, we calculated various indicators based on the confusion matrix. The confusion matrix is shown in Table 3:

**Table 3:** confusion matrix.

True label	Predicted label	
	Positive sample	Negative sample
Positive sample	TP	FN
Negative sample	FP	TN

Among them, TP (true positive) represents that a positive sample is predicted to be a positive sample; FP (false positive) represents that a negative sample is predicted to be a positive sample; FN (false negative) represents that a positive sample is predicted to be a negative sample; TN (true negative) represents a negative sample is predicted to be a negative sample. Several evaluation indicators can be obtained based on the confusion matrix. The commonly used indicators are accuracy (Acc), precision (P), recall (R), and F1 value. The specific calculation method is as follows:

26 $$Accuracy=\frac{TP+TN}{TP+FP+FN+PN}$$

27 $$Precision=\frac{TP}{TP+FP}$$

28 $$Recall=\frac{TP}{TP+FN}$$

29 $$\mathrm{F}1=\frac{2\times Precision\times Recall}{Precision+Recall}$$

In addition, the receiver operating characteristic curve (ROC) and the area under the ROC curve (AUC) were used to evaluate the algorithm.

### Analysis of MAD-FPC

This experiment was run on an Intel core i7-9750H@2.6 GHz processor, 16G memory, and Python 3.7.2 environment. The recognition results of the multi-classification experiments are counted according to the attack categories, which are Normal, DOS, Probe, U2R, and R2L. In terms of parameter selection, the fuzzy clustering hyperparameter *m* is set to 2, and the *C* value is set to 5.

#### Analysis of fuzzy clustering results

**Figure 4:**
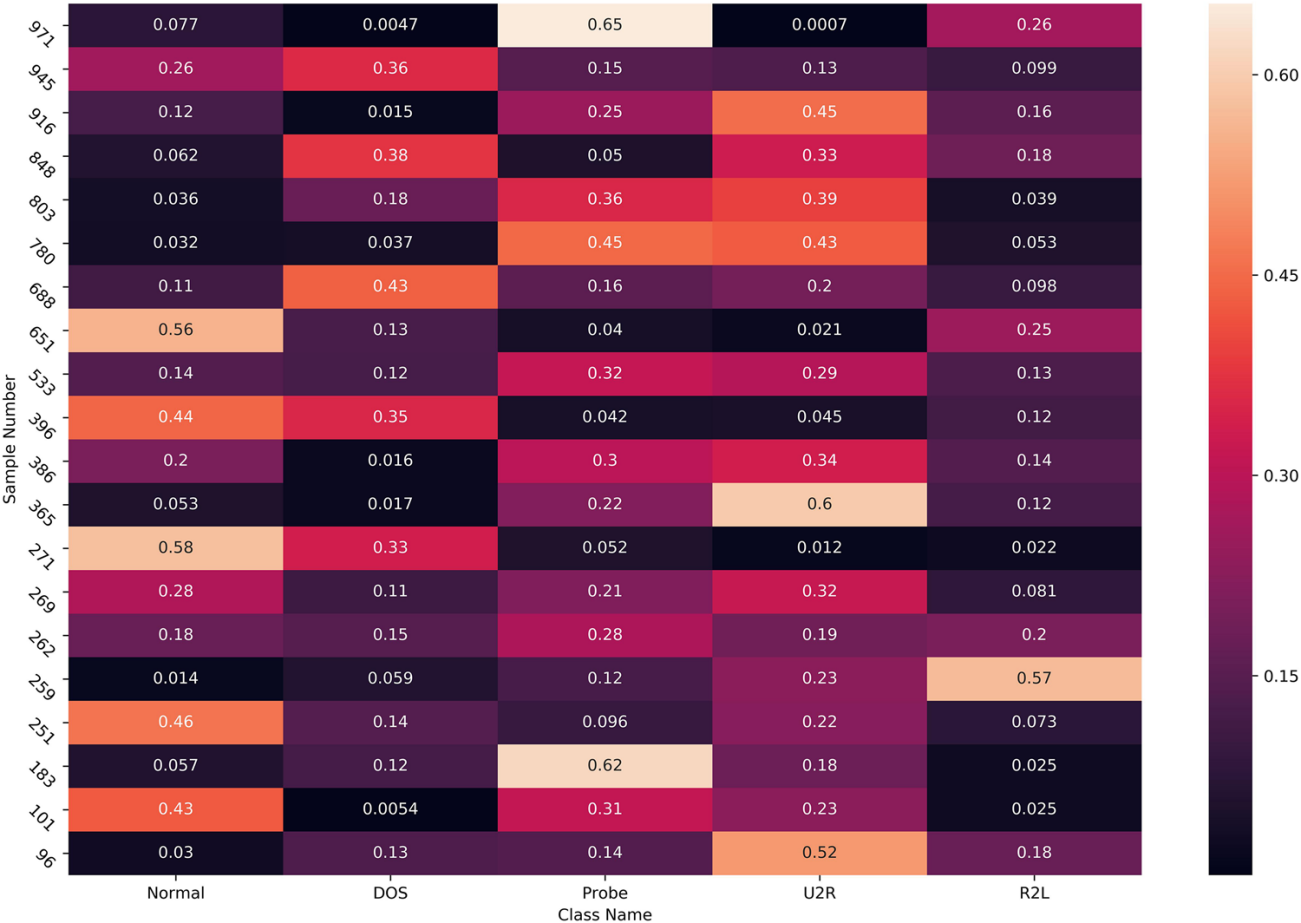
Examples of FPC algorithm results.

The fuzziness of the FPC algorithm is reflected in the fact that a sample can belong to multiple categories at the same time, and the value of the membership degree determines the magnitude of the correlation. The fuzzy clustering results are shown in Fig. 4. We randomly select several samples to display the membership degree. If the value of the membership degree is larger, the sample is more likely to belong to this class. The values of the membership degrees of each class corresponding to each sample are summed up to 1.

**Figure 5:**
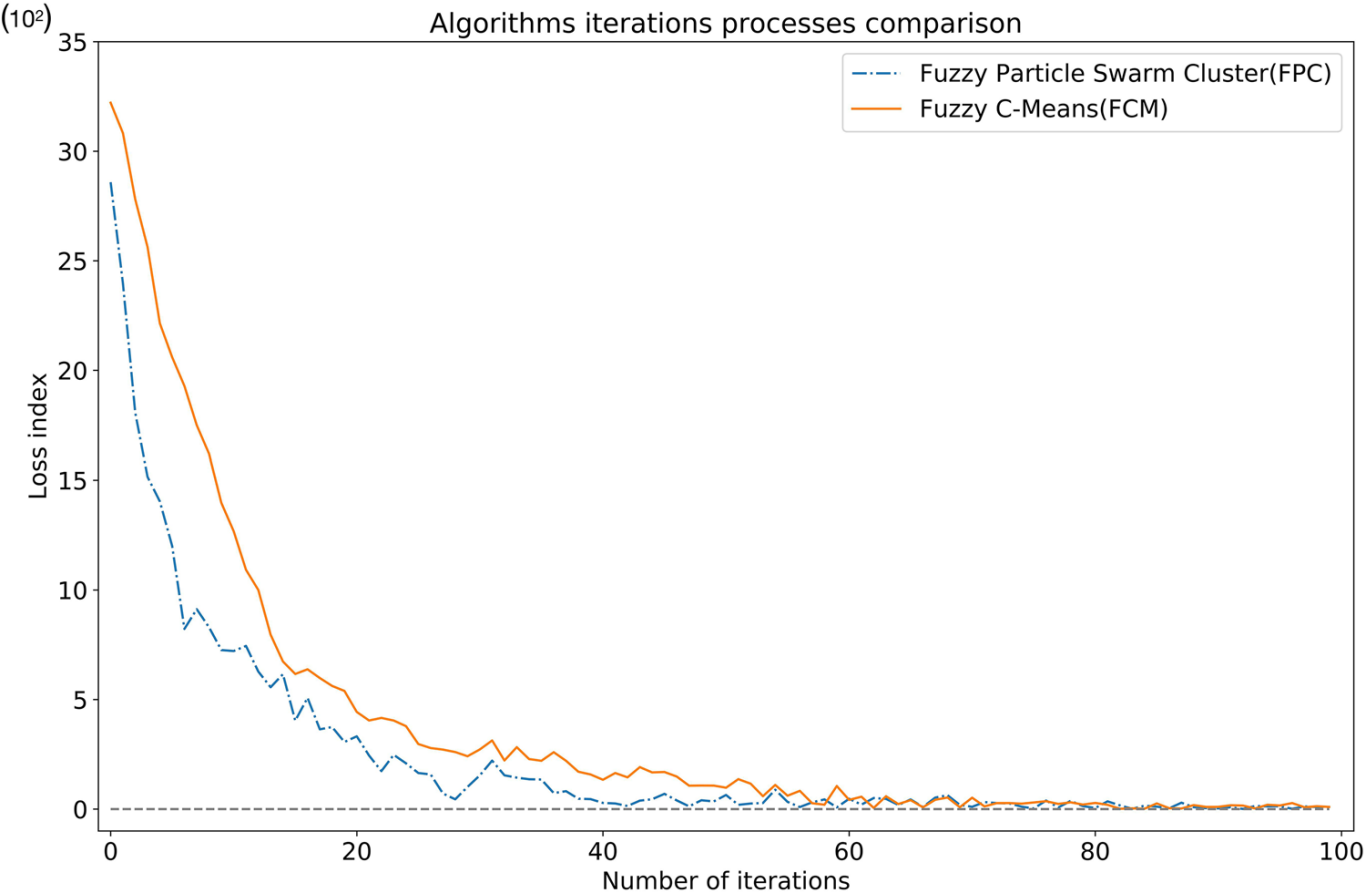
The change of loss index of the FPC algorithm and the FCM algorithm.

Since we use the particle swarm process to optimize the original fuzzy clustering algorithm, we pay special attention to the relationship between the number of iterations and the fitness value in the experiment. Because we redesigned the FPC algorithm's fitness function (formula 5), it is different from the fitness function of the FCM algorithm, especially in terms of changes in the difference. The absolute value of "Fitness Value" of the FCM algorithm is relatively large, while the absolute value of the "FitnessValue" of the FPC algorithm is relatively small, so it is difficult to compare the differences when the two graphs are placed together. In order to compare the iterative convergence speed of these two algorithms, we designed the "Loss Index" to dynamically measure the range of fitness values. The Y-axis coordinate stands for the loss value, equal to the current adaptation value minus the minimum adaptation value (the minimum changes dynamically if the current value is less than the minimum), and the X-axis coordinate represents the number of iterations. We run FPC and FCM 10 times with each iteration times set to 100 to get the Loss Value. Figure 5 shows the changes in the loss index during the execution of the algorithms. It can be seen from the figures that with the continuous increase in the number of iterations, the fitness values of the two algorithms both decrease and finally converges to a smaller interval. Compared with FPC, the convergence speed of the FCM algorithm is slower, and the optimal clustering effect can only be achieved at the 60th iteration. The FPC algorithm uses the particle swarm to find the center of the fuzzy clustering, so the convergence speed is faster than that of the FCM algorithm, and the better clustering effect has been achieved in the 23rd iteration. Near the 40th iteration, the best result on the training set was reached (representing the Loss index equals 0). As the program continues to execute, the particle swarm shows a certain instability causing the fitness value to jump up and down violently. The reason for this is that the learning rate is a fixed value, although it can converge at a fast speed in the early stage, the particles may cross the optimal position in the later stage when moving. However, on the whole, the performance of the FPC algorithm is still better than the FCM algorithm.

Due to the characteristics of the loss value, when the current value is less than the known minimum value, the global minimum Loss Value will be exchanged, resulting in a point where Loss Value = 0. Table 4 is the comparison of Loss Value in the iterative process.

**Table 4:** Comparison of loss value.

Number of iterations	The loss value of FPC (10^2^)	The loss value of FCM (10^2^)
0	28.58	32.21
1	23.96	30.83
2	18.07	27.80
3	15.16	25.63
4	14.04	22.15
5	12.01	20.60
6	8.22	19.28
7	9.12	17.50
8	8.29	16.19
9	7.25	13.97
10	7.21	12.68
11	7.44	10.92
12	6.27	10.00
13	5.56	7.95
14	6.15	6.73
15	4.03	6.16
16	5.08	6.38
17	3.64	5.98
18	3.74	5.62
19	3.06	5.39
20	3.32	4.43
21	2.44	4.04
22	1.73	4.16
23	2.47	4.04
24	2.08	3.78
25	1.64	2.97
26	1.58	2.78
27	0.71	2.71
28	0.44	2.60
29	1.01	2.41
30	1.54	2.72
31	2.21	3.13
32	1.54	2.21
33	1.43	2.82
34	1.36	2.28
35	1.34	2.20
36	0.73	2.59
37	0.81	2.20
38	0.47	1.70
39	0.45	1.57
40	0.28	1.33
41	0.25	1.64
42	0.14	1.44
43	0.38	1.91
44	0.45	1.67
45	0.69	1.69
46	0.39	1.48
47	0.12	1.06
48	0.40	1.07
49	0.35	1.07
50	0.64	0.97
51	0.19	1.36
52	0.25	1.16
53	0.27	0.58
54	0.88	1.10
55	0.33	0.61
56	**0.00**	0.83
57	0.30	0.26
58	0.44	0.20
59	**0.00**	1.05
60	0.47	0.42
61	0.20	0.56
62	0.50	**0.00**
63	0.46	0.58
64	0.18	0.23
65	0.44	0.40
66	**0.00**	**0.00**
67	0.52	0.42
68	0.63	0.52
69	0.19	**0.00**
70	**0.00**	0.52
71	0.32	0.12
72	0.27	0.27
73	0.26	0.27
74	0.11	0.24
75	**0.00**	0.30
76	0.39	0.36
77	**0.00**	0.23
78	0.37	0.31
79	0.13	0.20
80	0.05	0.28
81	0.35	0.19
82	0.17	**0.00**
83	**0.00**	0.03
84	0.15	**0.00**
85	0.11	0.25
86	**0.00**	0.04
87	0.29	**0.00**
88	0.08	0.18
89	**0.00**	0.09
90	**0.00**	0.10
91	0.08	0.17
92	**0.00**	0.16
93	0.14	**0.00**
94	0.12	0.19
95	0.12	0.16
96	**0.00**	0.27
97	0.11	0.08
98	0.09	0.13
99	0.11	0.09

Significant values are in bold.

It can be seen from Table 4 that the convergence speed of the FPC algorithm is significantly faster than that of the FCM algorithm, and after the number of iterations exceeds 60, the value of Loss Value is 0 for 11 times, which means that the optimal value of FPC Values are swapped 11 times. Meanwhile, FCM has only exchanged 7 times. Therefore, the particle swarm jumping behavior of FPC plays a very important role in the process of finding the optimal clustering result, which is also one of our innovations.

#### Analysis of anomaly detection results

**Figure 6:**
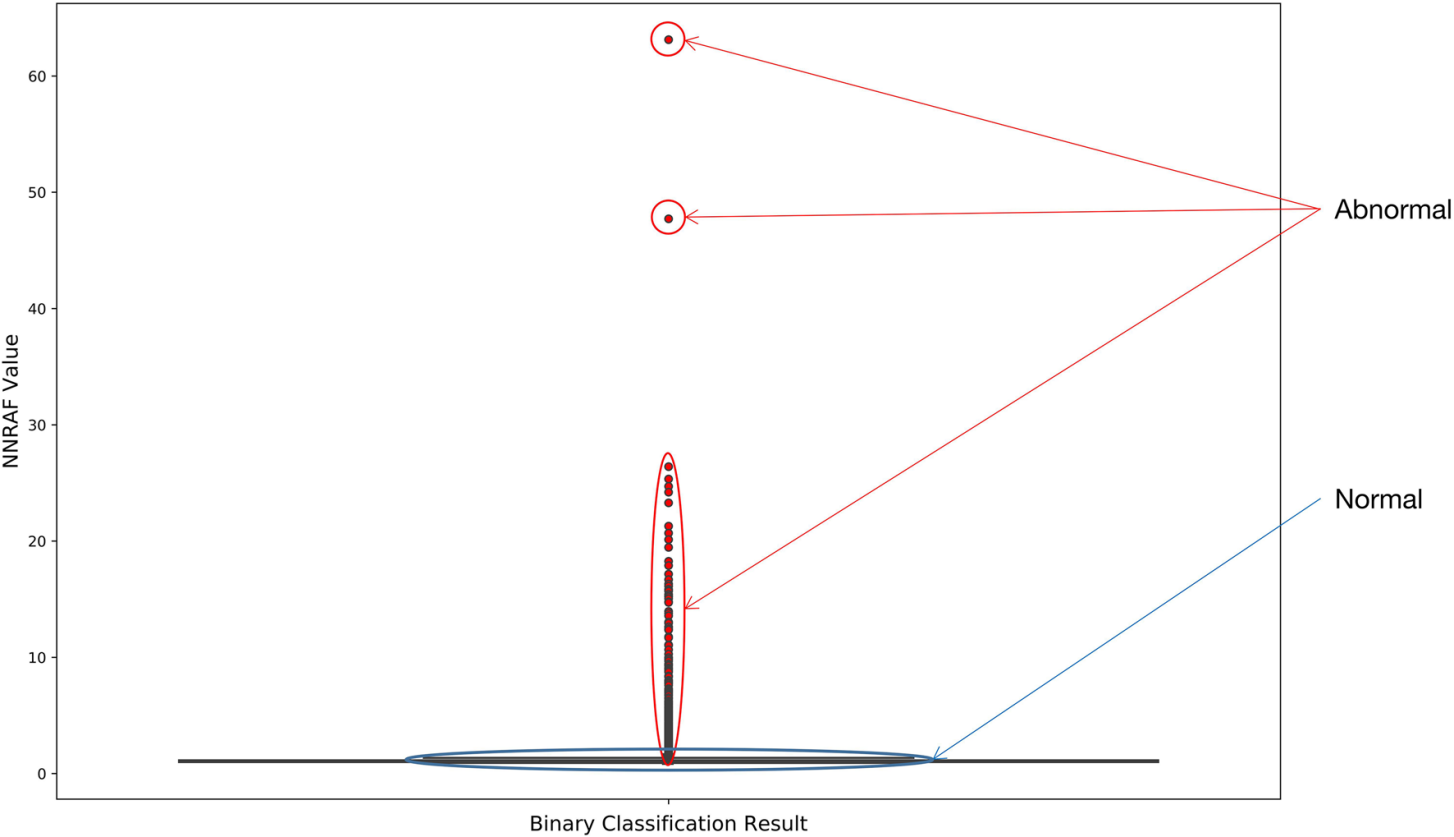
Anomaly detection results of NNRAF by using boxplot.

We compute the *NNRAF* for each sample and detect outliers in it using boxplot. In Fig. 6, the outliers detected by the boxplot have been marked in red, and we can see that a large number of data *NNRAF* values are relatively low, mainly concentrated within 3. The *NNRAF* values of abnormal samples also show a certain density trend, most of which are between 30 and 3, and the highest *NNRAF* value obtained by calculation is 63.13200726.

**Figure 7:**
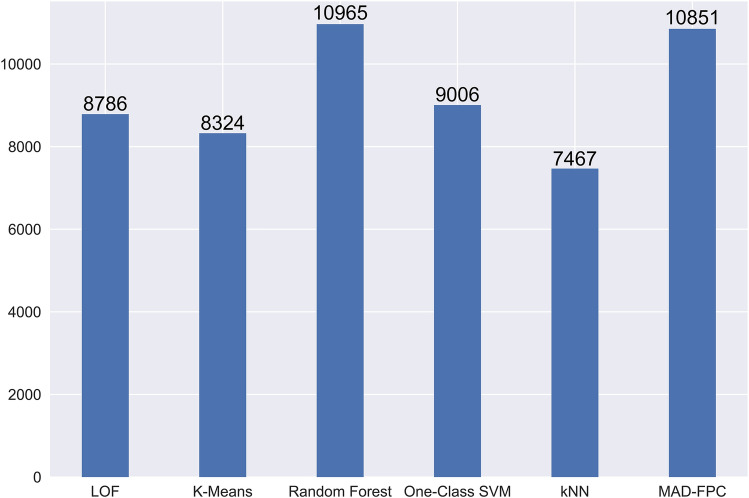
Comparison of abnormal detections in LOF, K-Means, Random Forest, One-Class SVM, kNN and the MAD-FPC.

We selected LOF, K-Means, Random Forest,One-Class SVM, kNN and the MAD-FPC algorithm we proposed to compare the anomaly detection effect. In terms of the number of abnormal behaviors detected, the number of abnormal behaviors detected by the algorithm proposed in this paper is roughly the same as that detected by the random forest algorithm. Among the 12,833 abnormal samples, a total of 10,851 non-Normal data were identified by MAD-FPC. The anomaly detection results in the binary classification of complete data are shown in Fig. 7.

#### Comparisons of evaluating performance

The detection result of the Normal class is to analyze the data set as a whole, regard DOS, Probe, U2R, and R2L as anomalies, and then judge the anomaly detection ability of the MAD-FPC algorithm. The multi-homed detection experiment is to first split the data set according to the class and then mix it with the Normal class data to judge the anomaly detection performance from normal samples. Table 5 shows the evaluation indicators of the 6 algorithms on multi-category data. The overall performance of the MAD-FPC algorithm is the best, and the random forest is slightly weaker. At the same time, we also noticed that due to the small number of samples in the U2R and R2L categories, the model might learn little knowledge in modeling these two types of data. The detection effect of the MAD-FPC algorithm on these two categories is weaker than that of other categories. Therefore, the problem of the small amount of sample data in the U2R and R2L categories affects the average value of the final evaluation index to a certain extent.

**Table 5:** Comparison of evaluation indexes.

Categories	Algorithms																							
	LOF				K-means				Random forest				One-class SVM				KNN				MAD-FPC			
	Acc	P	R	F1	Acc	P	R	F1	Acc	P	R	F1	Acc	P	R	F1	Acc	P	R	F1	Acc	P	R	F1
Normal	0.77	0.89	0.91	0.90	0.67	0.91	0.85	0.88	0.93	0.96	**0.96**	0.96	0.67	0.92	0.77	0.84	0.77	0.88	0.77	0.82	**0.95**	**0.98**	0.95	**0.96**
DOS	0.78	0.81	0.82	0.81	0.76	0.80	0.81	0.80	0.89	0.96	0.92	0.94	0.69	0.75	0.74	0.74	0.71	0.77	0.73	0.75	**0.94**	**0.98**	**0.96**	**0.97**
Probe	0.78	0.83	0.85	0.84	0.70	0.75	0.71	0.72	0.74	0.84	**0.94**	0.89	0.58	0.66	0.71	0.69	0.78	0.81	0.75	0.78	**0.94**	**0.96**	0.82	0.88
U2R	0.72	0.79	**0.88**	0.83	0.70	0.79	0.79	0.79	**0.85**	0.85	0.82	0.84	0.64	0.73	0.86	0.79	0.66	0.71	0.68	0.70	**0.85**	**0.91**	**0.88**	**0.89**
R2L	0.68	0.74	0.86	0.79	0.68	0.76	0.80	0.78	0.92	0.95	**0.89**	0.92	0.68	0.80	0.80	0.80	0.65	0.69	0.73	0.71	**0.93**	**0.98**	**0.89**	**0.93**
Average	0.75	0.81	0.86	0.84	0.70	0.80	0.79	0.79	0.87	0.91	**0.91**	0.91	0.65	0.77	0.78	0.77	0.71	0.77	0.73	0.75	**0.92**	**0.96**	0.90	**0.93**

Significant values are in bold.

**Figure 8:**
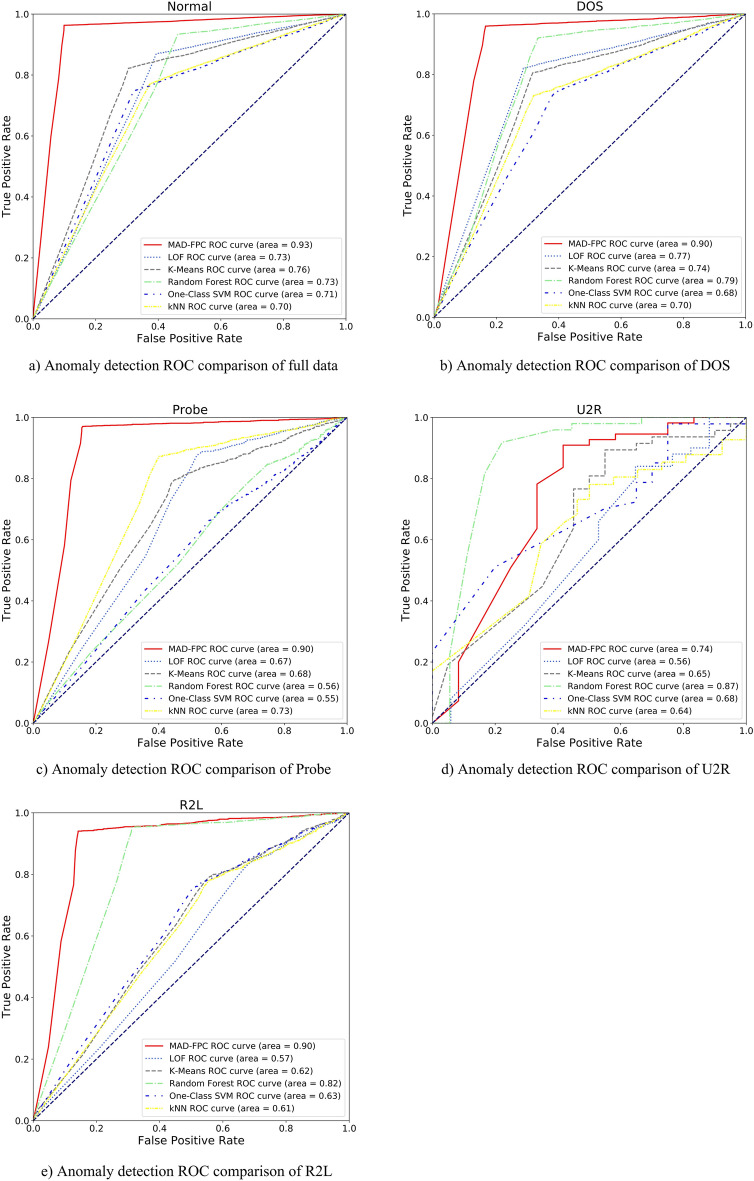
Comparison of ROC curves of different algorithms.

Combined with Fig. 8, although the number of outliers detected by the MAD-FPC algorithm is not as high as that detected by the random forest algorithm, its overall evaluation index is better than the random forest algorithm, so the MAD-FPC algorithm is more suitable for detecting abnormal data.

From the ROC curve of each subgraph in Fig. 8, the MAD-FPC algorithm performs best in the anomaly detection experiment of the full dataset. The improved fuzzy clustering based on BF-IEF optimization technology can better distinguish the behavior of various entities, so as to obtain a better fuzzy clustering effect. Moreover, in the classification anomaly detection test, the diversity of entity categories is reduced, and the advantage of the MAD-FPC algorithm in the use of multi-homed attribution information is reduced, so the detection advantage presented is not as large as that in the full data set anomaly detection test. Compared with the traditional LOF algorithm, the MAD-FPC algorithm, which has been improved by the attribute of the multi-homed of the entity sample, calculates the abnormal factor of more dimensions and can detect more abnormal points.

In addition, we also tested the real-time recognition ability of the MAD-FPC algorithm using a simulated data set, and the specific results are shown in Table 6. The structure of the simulated data is made by imitating NSL-KDD data, and the simulated attack is carried out in the virtual environment of the network security laboratory. Set the centroid of the training data set to the fuzzy cluster centroid of the real-time recognition experiment to initialize the experimental parameters. When the data flow starts to attack, the degree of membership of the sample is directly calculated, then the *NNRAF* of the sample is calculated, and finally, the 3σ Rule based on the boxplot is used for anomaly detection. Since the training results are used directly, the detection efficiency can reach real-time. The experiment shows that the MAD-FPC algorithm is also superior to other algorithms in small-batch online detection, and is suitable for all-weather deployment detection as an incremental detection model for entity behavior.

**Table 6:** Comparison of evaluation indexes.

	LOF	K-means	Random forest	One-class SVM	kNN	MAD-FPC
Abnormal recognition rate	91.13%	90.55%	92.06%	89.32%	80.16%	94.88%

From the experimental results, the overall performance of the MAD-FPC algorithm for entity behavior anomaly detection is better than the LOF, K-Means, Random Forest, One-Class SVM, and kNN algorithms, especially in the abnormal detection of the Normal category. In practical applications, the main function of entity behavior analysis is to find abnormal samples in user and entity behavior data, so the MAD-FPC algorithm is more suitable for anomaly detection of current entity behavior than the other three algorithms.

## Conclusion

With the continuous development of network technology and the continuous expansion of enterprise business scale, the network security threats that enterprises face have become increasingly complex and changeable. Therefore, studying entity behavior anomaly detection methods can further reduce the network security threats enterprises face. Inspired by the above discussion, in this work, we proposed an anomaly detection algorithm based on fuzzy clustering and multi-homed cluster attribution, which was mainly used in entity behavior analysis. Firstly, an entity behavior fuzzy clustering algorithm is designed based on the behavior measurement method using differences between entity behavior and other entity behavior. The membership matrix between the entity behavior and the category is obtained through particle swarm optimization and correlation calculation, which solves the problem of a single analysis angle of the deterministic clustering algorithm. Then, based on the membership degree matrix, a multi-homed attribution anomaly detection algorithm is designed to analyze the relative structure of the entity's behavior in each class and judge the anomaly of the point. The MAD-FPC algorithm solves the shortcomings of the traditional anomaly detection algorithm's "hard division" of entity behavior. It obtains more accurate detection results by calculating the degree of an anomaly in different classes.

In the future, we will continue UEBA research and establish a network security protection model to identify unknown abnormal traffic. Deploying this model in cybersecurity protective equipment increases the influence of our research findings not only in the industrial circles but also in the academic sector.

## Data Availability

NSL-KDD is a well-known public data set in the cyber security field. The data is openly available at https://www.unb.ca/cic/datasets/nsl.html. The more specific feature engineering approach that supports the findings of this study is available from the corresponding author upon reasonable request.
